# Scaphoid Fat Stripe Sign: Is It a Reliable Radiological Sign of Scaphoid Fracture in Children?

**DOI:** 10.3390/children12010086

**Published:** 2025-01-13

**Authors:** Pavle Manic, Stéphanie Schizas, Pierre-Yves Zambelli, Eleftheria Samara

**Affiliations:** University Hospital of Lausanne, 1011 Lausanne, Switzerland; pavle.manic@unil.ch (P.M.); stephanie.schizas@unil.ch (S.S.); pierre-yves.zambelli@chuv.ch (P.-Y.Z.)

**Keywords:** scaphoid fat pad sign, scaphoid fracture, radiological diagnosis

## Abstract

Objectives: The scaphoid fat pad stripe (SFS) is a radiological sign first described in 1975 as a line of relative lucency lying parallel to the lateral border of the scaphoid, with slight convexity toward it, and it is optimally demonstrated on postero-anterior and oblique views with ulnar deviation of the carpus. The obliteration or displacement of this line is commonly present in acute fractures of the scaphoid, radial styloid process, and proximal first metacarpus. The aim of this observational study is to investigate the supportive value of the fat stripe sign (SFS) in the diagnosis of scaphoid fractures in the pediatric population. Methods: This is a monocentric, retrospective study of all patients referred to the Pediatric Traumatology Unit of a tertiary hospital from the Emergency Department with clinical suspicion of scaphoid fracture without visible fracture in the initial X-ray. Radiological reports for CT and MRIs were recorded, and the initial X-rays were blindly reviewed by a pediatric orthopedic fellowship-accredited surgeon for the presence of an abnormal scaphoid fat pad stripe sign and the presence of a fracture line in the initial X-rays. Results: The results of the blind interpretation of the initial X-rays for the fat stripe sign showed 86% sensitivity and 58% specificity, with the negative predictive value reaching 92%. Conclusions: The scaphoid fat stripe sign can be used as an adjacent in the diagnosis of an occult scaphoid fracture in children or adolescents. Its high negative predictive value, if confirmed in larger studies, can be an element used to exclude scaphoid fracture and consequently avoid unnecessary immobilizations and health costs.

## 1. Introduction

Wrist trauma is a frequent occurrence among children seeking care in the emergency department. Scaphoid fractures are the most common fractures of the carpus [1] and, although rare [2,3], there has been an increasing incidence in past years, which has been attributed to earlier and increased participation in youth sports [4,5]. Most pediatric scaphoid fractures occur in the 11- to 13-year-old age group, when ossification is complete enough to establish the location of the fracture [6,7]. In contrast to adults, there is a predominance of fractures in the distal pole of the scaphoid in children [3,8]. The prevalence of distal pole fractures increases the likelihood of tenderness over the scaphoid tuberosity compared with the waist fractures, which cause pain to palpation within the anatomic snuffbox [3]. Physicians with a clinical suspicion of a scaphoid injury should ask for posterior–anterior (PA), lateral, and oblique radiographs of the wrist. In addition, special scaphoid views, consisting of anterior–posterior (AP) wrist views with bent fingers and with the wrist in 24° to 45° of supination, can enhance the visualization of the scaphoid. These radiographs place the scaphoid parallel to the film and reveal the scaphoid in its full size [9]. Scaphoid fractures are often undetectable on initial radiographs, especially in the acute setting [10,11]. They can be initially radiographically occult in 12.5% to 37% of cases [12,13]. Soft tissue abnormalities on radiological examination can be useful in evaluating injury to various regions. Although the posterior fat pads of the elbow joint, as an indicator of underlying fracture in children following injury [5], have been widely accepted as a reliable radiological sign, to our knowledge there is no study published on the reliability and supportive value of the fat stripe sign in the pediatric population.

**Figure 1:**
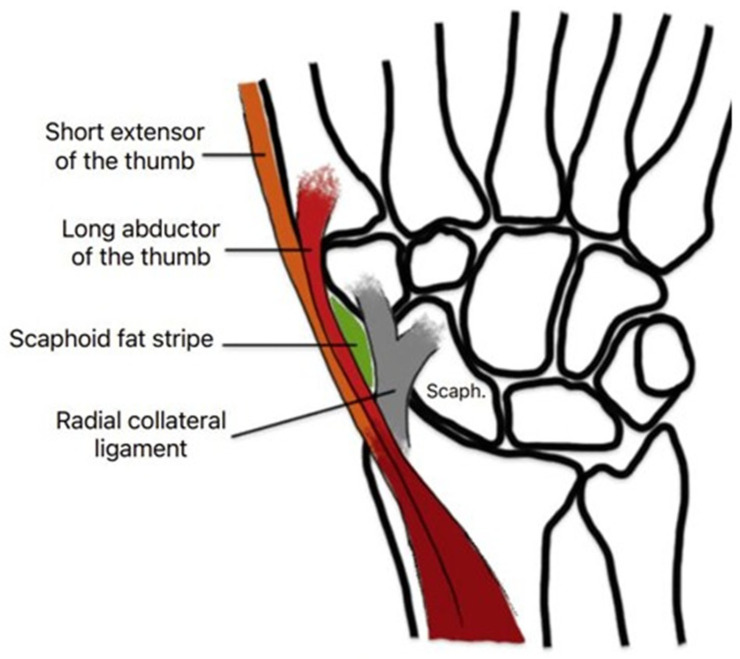
A diagram of the anatomical region of the scaphoid fat stripe.

In 1975, Terry and Ramin firstly described the scaphoid fat pad stripe (SFS) in adult wrist trauma. They described a line of relative lucency lying parallel to the lateral border of the scaphoid, with slight convexity toward it, that is optimally demonstrated on postero-anterior and oblique views with ulnar deviation of the carpus. Anatomically, the “scaphoid fat stripe” (SFS) was found to correspond the highly radiolucent common tendon sheath of the extensor pollicis brevis and the abductor pollicis longus muscles [14] (Figure 1). The obliteration or displacement of this line corresponds to blood or oedema fluid accumulation in a small collection of fatty tissue wedged between the radial collateral ligament of the wrist joint and the synovial sheaths of the abductor pollicis longus and extensor pollicis brevis tendons, which are inserted into the radial aspect of the bases of the first metacarpal and proximal phalanx of the thumb, respectively [10]. The obliteration or displacement of this line is commonly present in acute fractures of the scaphoid, radial styloid process, and proximal first metacarpus. A positive fat stripe sign serves to alert the physician that an underlying fracture is likely [10,15].

The aim of this observational study is to investigate the supportive value of the fat stripe sign (SFS) in the diagnosis of scaphoid fractures in the pediatric population.

## 2. Materials and Methods

Between January 2011 and December 2020, we obtained patient details by a review of a regional Picture Archiving and Communications System (PACS), identifying all patients who had received scaphoid X-rays and CT or MRI of the wrist and hand in the Pediatric Traumatology Unit of the Children University Hospital of Lausanne in Switzerland during this period. These were subsequently reviewed to identify patients who had undergone trauma and were referred from the Emergency Department to the Pediatric Orthopaedics and Traumatology Unit. The inclusion criteria were (1) skeletal immaturity as determined by the presence of open physes; (2) referral to children trauma and orthopedics departments due to tenderness either in the anatomical snuff box (ASB) or tenderness over the scaphoid tubercle (ST), pain on longitudinal compression of the thumb (LC) and the range of thumb movement (TM) as per the institutional protocol of referral; (3) treatment with antebrachial immobilization and repeat radiographs or MRI in 10 to 14 days, as per the standard institutional protocol of treatment for suspected scaphoid bone fractures. Pathological fractures, multiply injured patients, and patients who had previously undergone wrist surgical fixation were all excluded from the study. After approval by the Swiss Ethics Committee (CER-VD 2022-01831), the records were reviewed for information regarding sex, mechanism of injury, and fracture type. Radiological reports for CT and MRIs were recorded and the initial X-rays were blindly reviewed by a first-year resident of pediatric surgery and a pediatric orthopedic fellowship-accredited surgeon for the presence of an abnormal scaphoid fat pad stripe sign and the presence of a fracture line in the initial X-rays. The inter-rater agreement of their ranks was checked, as well as the intra-rater agreement of the pediatric orthopedic surgeon on her documentation at different time points, blindly. Due to the low sample size, we did not test the intraclass correlation coefficient (ICC) as a reliability index.

## 3. Results

**Figure 2:**
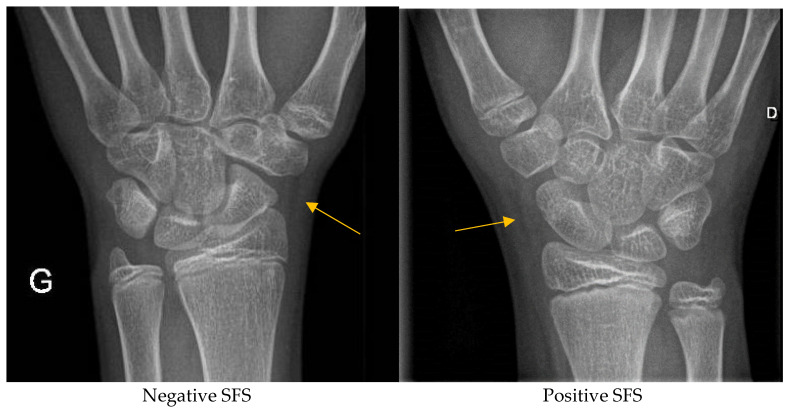
Examples of negative and positive SFS sign.

In the 10-year period, forty-two children were eligible for this study. Of these, only 26 could be reached and agreed to the reuse of the data for research purposes. The mean age was 14 years and 2 months, with predominantly the male sex represented at 65.4% (17/26). All patients had at least two clinical signs indicative of scaphoid fracture when referred to the pediatric orthopedics clinic. In seven patients (27%), the definitive diagnosis was scaphoid fracture (male/female: 6/1) and in 19 (73%) this diagnosis was discarded. Among the seven patients positive for a fracture, four were diagnosed at the day of trauma by plain X-rays, one with a CT performed at day 0, and the other two with MRI after 10 days of immobilization due to clinical suspicion. Among the 19 patients susceptible to a scaphoid fracture, all were immobilized for an average of 11 days, and the definitive diagnosis was given with an MRI in 12 patients (63.1%). Of them, only one patient underwent prolonged immobilization due to the detection of an occult distal radius fracture invisible in the plain films. Seven patients were asymptomatic after immobilization, and no visible fracture in the plain films excluded scaphoid fracture definitively. The results of the blind interpretation of the initial X-rays for the fat stripe sign and the definitive diagnosis are summarized in Table 1 for both observers. In () are documented the responses of the first-year resident with basic training in pediatric traumatology. There is a high consistency in the detection of SFS by both observers, with absolute agreement in the negativity of the sign when there was no fracture. The intra-rater agreement test from the pediatric orthopedic surgeon was absolute at two different time points, blindly, as well. True positive results were frequent, with 6/7 X-rays for both observers, in contrast to true negatives, which were less accurate and fairly inconsistent between the observer groups (11/19 and 8/19). Examples of positive and negative SFS are given in Figure 2.

## 4. Discussion

The optimal management and treatment of clinical scaphoid fractures in pediatric patients remain unknown. Standard treatment involves immobilization and repeat radiographs in 10 to 14 days, with the belief that the delay allows for bony resorption adjacent to the fracture site, making the fracture visible [16,17,18,19]. Although this approach remains an accepted option for treatment, it may result in unwarranted immobilization, with a negative impact on school life, unnecessary imaging, and multiple clinical visits incurring health care costs and a negative impact on family life [16].

To our knowledge, this is the first study to investigate the presence of the fat stripe sign in children and adolescents with a clinical suspicion of scaphoid fracture. Relatively limited also is the literature on the adult population, where the SFS is considered a poor predictor of a scaphoid fracture, even though a positive scaphoid fat pad sign indicates that an underlying fracture is likely [10,20]. Several studies in adults have suggested that the use of early MRI is cost-effective in identifying and managing these fractures, but no similar studies have been conducted in the pediatric population [16]. The incorporation of an early MRI into a treatment protocol may be inconvenient for the majority of young patients, costly, and maybe unnecessary, since we do not know if the risk of nonunion and AVN in occult scaphoid fractures is similar to the risk in scaphoid fractures that are readily apparent on initial X-ray.

This study has the inherent limitation of being a retrospective review with a limited population, which limits the ability for powerful statistical analysis. Despite its limitations, it is the first study studying the SFS sign only in the pediatric population, and we reveal the potential supportive value of the SFS radiological sign in the early diagnosis of scaphoid fractures in children and adolescents. This observational study aims to reveal the potential utility of a “forgotten” radiological sign and motivates clinical research. Certainly, future prospective multicenter studies are needed to answer whether the fat stipe sign has a high sensitivity and specificity and what its predictive value is. Inter-observer reliability should be checked in future studies, as this radiological sign needs some training to be easily detected, and the learning curve is unknown. Nowadays, with the implementation of artificial intelligence in diagnostic radiology, this may be a feature to be added in training data in AI models if proven to be of clinical interest as well.

## 5. Conclusions

The scaphoid fat stripe sign can be used as an adjacent in the diagnosis of an occult scaphoid fracture in children or adolescents. Its high negative predictive value, if confirmed in larger studies, can be a serious element to exclude scaphoid fracture and consequently avoid unnecessary immobilizations, school absenteeism, and health costs.

## Figures and Tables

**Table 1:** Incidence of scaphoid fracture and scaphoid of fat stripe sign as documented from both observers. In () are incidences of first-year resident.

		Definitive Diagnosis: Scaphoid Fracture		
		Fracture	No Fracture	Total
Scaphoid Fat Stripe Sign (First X-Ray)	Positive	6 (6)	8 (11)	14 (16)
	Negative	1 (1)	11 (8)	12 (10)
	Total	7 (7)	19 (19)	26 (26)

## Data Availability

The data presented in this study are available on request from the corresponding author. The data are not publicly available due to privacy and ethical permission.

## References

[1] Wulff R.N., Schmidt T.L. (1998). Carpal fractures in children. J. Pediatr. Orthop..

[2] Goddard N. (2005). Carpal fractures in children. Clin. Orthop. Relat. Res..

[3] Elhassan B.T., Shin A.Y. (2006). Scaphoid fracture in children. Hand Clin..

[4] Van Tassel D.C., Owens B.D., Wolf J.M. (2010). Incidence estimates and demographics of scaphoid fracture in the U.S. population. J. Hand Surg. Am..

[5] Lempesis V., Rosengren B.E., Landin L., Tiderius C.J., Karlsson M.K. (2019). Hand fracture epidemiology and etiology in children-time trends in Malmo, Sweden, during six decades. J. Orthop. Surg. Res..

[6] Mussbichler H. (1961). Injuries of the carpal scaphoid in children. Acta Radiol..

[7] Greene M.H., Hadied A.M., LaMont R.L. (1984). Scaphoid fractures in children. J. Hand Surg. Am..

[8] Ahmed I., Ashton F., Tay W.K., Porter D. (2014). The pediatric fracture of the scaphoid in patients aged 13 years and under: An epidemiological study. J. Pediatr. Orthop..

[9] Bohler L., Trojan E., Jahna H. (2003). The results of treatment of 734 fresh, simple fractures of the scaphoid. J. Hand Surg. Br..

[10] Banerjee B., Nashi M. (1999). Abnormal scaphoid fat pad: Is it a reliable sign of fracture scaphoid. Injury.

[11] Carver R.A., Barrington N.A. (1985). Soft-tissue changes accompanying recent scaphoid injuries. Clin. Radiol..

[12] Christodoulou A.G., Colton C.L. (1986). Scaphoid fractures in children. J. Pediatr. Orthop..

[13] Nafie S.A. (1987). Fractures of the carpal bones in children. Injury.

[14] Corfitsen M., Christensen S.E., Cetti R. (1989). The anatomical fat pad and the radiological “scaphoid fat stripe”. J. Hand Surg. Br..

[15] Terry D.W., Ramin J.E. (1975). The navicular fat stripe: A useful roentgen feature for evaluating wrist trauma. Am. J. Roentgenol. Radium Ther. Nucl. Med..

[16] Karir A., Huynh M.N., Carsen S., Smit K., Cheung K. (2022). Management and Outcomes of Clinical Scaphoid Fractures in Children. Hand.

[17] Parvizi J., Wayman J., Kelly P., Moran C.G. (1998). Combining the clinical signs improves diagnosis of scaphoid fractures. A prospective study with follow-up. J. Hand Surg. Br..

[18] Grover R. (1996). Clinical assessment of scaphoid injuries and the detection of fractures. J. Hand Surg. Br..

[19] Adams J.E., Steinmann S.P. (2007). Acute scaphoid fractures. Orthop. Clin. N. Am..

[20] Schunk K., Weber W., Strunk H., Regentrop H., Thelen R., Schild H. (1989). Traumatology and diagnosis of scaphoid fracture. Radiologe.

